# Adapting the Diabetes Prevention Program for Older Adults: Descriptive Study

**DOI:** 10.2196/45004

**Published:** 2023-08-29

**Authors:** Jeannette M Beasley, Emily A Johnston, Denisa Costea, Mary Ann Sevick, Erin S Rogers, Melanie Jay, Judy Zhong, Joshua Chodosh

**Affiliations:** 1 Department of Nutrition and Food Studies New York University Steinhardt School of School of Culture, Education, and Human Development New York, NY United States; 2 Department of Medicine New York University Grossman School of Medicine New York, NY United States; 3 Department of Population Health New York University Grossman School of Medicine New York, NY United States; 4 VA New York Harbor Healthcare System New York, NY United States

**Keywords:** aging, diabetes prevention program, nutrition, diet, physical activity, weight loss, weight, exercise, diabetes, prevention, diabetic, ageing, older adult, online intervention, digital intervention, virtual delivery, lifestyle coach, group-based intervention

## Abstract

**Background:**

Prediabetes affects 26.4 million people aged 65 years or older (48.8%) in the United States. Although older adults respond well to the evidence-based Diabetes Prevention Program, they are a heterogeneous group with differing physiological, biomedical, and psychosocial needs who can benefit from additional support to accommodate age-related changes in sensory and motor function.

**Objective:**

The purpose of this paper is to describe adaptations of the Centers for Disease Control and Prevention’s Diabetes Prevention Program aimed at preventing diabetes among older adults (ages ≥65 years) and findings from a pilot of 2 virtual sessions of the adapted program that evaluated the acceptability of the content.

**Methods:**

The research team adapted the program by incorporating additional resources necessary for older adults. A certified lifestyle coach delivered 2 sessions of the adapted content via videoconference to 189 older adults.

**Results:**

The first session had a 34.9% (38/109) response rate to the survey, and the second had a 34% (30/88) response rate. Over three-quarters (50/59, 85%) of respondents agreed that they liked the virtual program, with 82% (45/55) agreeing that they would recommend it to a family member or a friend.

**Conclusions:**

This data will be used to inform intervention delivery in a randomized controlled trial comparing in-person versus virtual delivery of the adapted program.

## Introduction

### Background

Without adopting healthier lifestyles, 15% to 30% of people living with prediabetes will develop type 2 diabetes within 5 years [1]. Moreover, diabetes in adults aged 65 years and older is connected to higher risks of complications and negative side effects compared to diabetes in younger age groups [2,3]. Therefore, it is important to implement public health programs tailored toward preventing diabetes among older adults. The seminal *Diabetes Prevention Program* (DPP) randomized controlled trial demonstrated that people with prediabetes who participate in these programs can decrease their risk of developing type 2 diabetes by 58% (or 71% for those aged >60 years) [2].

The “older adult” demographic consists of persons who are very fit, very frail, or healthy; those experiencing age-related health issues; persons living alone or in communities, assisted care facilities, or nursing homes; and persons with cognitive impairment and functional disabilities. This is a heterogenous group, and a tailored intervention that includes coaching and social support is particularly relevant to individuals at this stage of life. There is some concern with promoting weight loss in older adults due to the risk that they may also see significant decreases in fat-free mass and bone density with weight loss, compared to their younger adults (aged 25-45 years) and middle-aged adult counterparts (46-54 years) [4]. Intervention adaptations should be carefully planned to emphasize small changes and slow, steady weight loss.

Older adults with impaired mobility have a higher prevalence of obesity and obesity-related chronic conditions [5]. During lifestyle interventions, these participants encounter numerous barriers including health and mental health issues, transportation, and functional limitations [5]. Furthermore, Medicare beneficiaries face challenges in DPP implementation, especially with a lack of awareness from primary care providers. Medicare beneficiaries also express concerns about the ability to meet Centers for Disease Control and Prevention (CDC) program recognition requirements based on session attendance and achieving weight loss goals of 5% or more [6,7]. Barriers such as these require responsive adaptations. Thus, differing physiological compositions, biomedical needs, and psychosocial challenges among older adults require an individualized approach to diabetes prevention [5].

We previously conducted a 6-week pilot of the DPP among older adults to assess acceptability and feasibility [7]. Suggestions for improvement from this pilot included improving the sound quality of the connection between the health coach and participants, increasing the amount and depth of the nutrition information presented, and adapting materials for older adults. Program components added and revised to address this feedback include (1) providing closed captioning on all videos and personal amplifiers (PockeTalkers) to improve hearing in a group during intervention sessions; (2) incorporating additional resources regarding local programs that offer opportunities to access fruits, vegetables, and other healthy foods; and (3) accompanying each session with a brief video pertaining to the needs of older adults. These changes were made prior to the implementation pilot.

### Objectives

The purposes of this paper are to (1) describe adaptations of the successful National DPP’s PreventT2 lifestyle change program into the *Bringing the Diabetes Prevention Program to Geriatric Populations* (BRIDGE) randomized controlled trial, comparing the effectiveness and implementation of virtual versus in-person delivery of the DPP within a large health care system [8]; and (2) describe the results of a pilot of the adapted materials.

## Methods

### Resource Adaptations

The CDC’s National DPP is a partnership of public and private organizations functioning to prevent or slow type 2 diabetes [9]. The 2021 PreventT2 lifestyle change program is an evidence-based program focused on helping participants make positive lifestyle changes such as healthier eating, reducing stress with coping strategies, and becoming more physically active. Throughout the year-long program, participants attend 22 group sessions with a trained lifestyle coach with the goal of losing 5% to 7% of their body weight by implementing the behavior change strategies they discuss and work through together.

The study team evaluated the resources provided by the DPP for each session and topic. This initiated a process of DPP adaptation to incorporate additional resources to help older adults achieve intervention goals. Adaptations were based on feedback from a previous 6-week-long DPP pilot implemented by the study team [7], in addition to review by the multidisciplinary study team of dietitians (JMB and EAJ), physicians (JC and MJ), and experts in behavior change (MAS and ESR).

Certain sessions include interactive online modules that participants can complete on their own. The additional resources, including those related to nutrition, physical activity, stress management, and emotional support are summarized in Table 1. The program resources were compiled into a website that participants can easily access for free, in addition to the information presented in the group intervention sessions [10].

Additionally, participants were given specific instructions on how to use technology resources, including cell phone apps to track energy intake and physical activity. Videos include transcripts and closed captioning to increase information access for those with hearing loss.

**Table 1:** Enhanced Diabetes Prevention Program (DPP) curriculum.

Module	Additional resources [10]
Module 1: Introduction to the Program	Grocery shopping list apps (eg, AnyList or Mealime)
Module 2: Get Active to Prevent T2	Video resource—tips for exercising with limited mobility Video resource—common wheelchair injuries and prevention ADA^a^: injury free exercise Silver Sneakers^b^
Module 3: Track Your Activity	VA^c^ video resource—setting daily activity goals Calorie tracker how-to guides (eg, HealthWatch360)
Module 4: Eat Well to Prevent T2	USDA^d^ Food Guide Apps: FoodKeeper and Food Safety Department of Health and Human Services’ Cold Food Safety Chart—provides tips and information on food storage after preparing food Video resource—eating healthy to prevent kidney disease
Module 5: Track Your Food	VA video resource—reminders and record keeping Food tracker how to guides (eg, HealthWatch360)
Module 6: Get More Active	VA video resource—basics of PA^e^ Multiple PA videos
Module 7: Energy In, Energy Out	Video resource—Prevent T2 curriculum handouts: Energy In, Energy Out VA video resource—food composition and dietary guidelines
Module 8: Eating to Support Your Health Goals	VA video resources—Eating Wisely at Home and Managing Hunger CDC^f^ article—Improving Your Eating Habits NIH^g^ article—Healthy Meal Planning: Tips for Older Adults
Module 9: Manage Stress	NIH video resource—GREAT: Helpful Practices to Manage Stress NIH article—5 Things You Should Know About Stress VA video resource—Stress
Module 10: Eat Well Away From Home	NIDDK^h^ video resource—Healthy eating at family gatherings USDA article—Dine Out/Take Out
Module 11: Managing Triggers	VA video resource—What is Self-Management? YouTube—Changing the Things Around You
Module 12: Stay Active to Prevent Type 2	NCHPAD^i^ video—Intro to Exercise Episode 8: How to Make Exercising Fun Multiple PA videos
Module 13: Take Charge of Your Thoughts	VA video resource—Changing Your Thinking about Food, Exercise, and Yourself YouTube—Take Control with Positive Thinking
Module 14: Get Back on Track	VA video resource—Putting Knowledge Into Action
Module 15: Get Support	HealthClips Online—Finding the Support You Need When You Have Prediabetes + Transcript Testimonials from DPP participants
Module 16: Stay Motivated to Prevent Type 2	VA video resource—I Choose Health: Goal Setting
Module 17: When Weight Loss Stalls	VA video resource—Coping with Weight Plateaus
Module 18: Take a Movement Break	NIH video resource—The Emotional Benefits of Exercise Physical Activity Break—Overhead Stretch: VA Move! Physical Activity Break videos from VA Move!
Module 19: Keep Your Heart Healthy	YouTube—What is Cholesterol? CDC—Prevent Heart Disease NIH—Use Herbs and Spices Instead of Salt VA video resource—Heart Healthy Cholesterol
Module 20: Shop and Cook to Prevent T2	VA Move!—Eating Well on a Budget NIDDK—Eat Healthy Even When You Don’t Have Time to Cook Hunter College NYC^j^ Food Policy Center article—Cooking Demo Instructions: Fresh Taste for Seniors
Module 21: Get Enough Sleep	VA video resource—I Choose Health – Sleep NIH—A Good Night's Sleep | National Institute on Aging CDC article—Sleep and Chronic Disease
Module 22: Prevent Type 2 – For Life!	NIH CDC video—Preventing Type 2 Diabetes NIDDK video—Small Steps, Big Difference: Preventing diabetes is within your reach

^a^ADA: American Diabetes Association.

^b^Physical activity classes designed for older adults.

^c^VA: Veterans Association.

^d^USDA: United States Department of Agriculture.

^e^PA: physical activity.

^f^CDC: Centers for Disease Control and Prevention.

^g^NIH: National Institute of Health.

^h^NIDDK: National Institute of Diabetes and Digestive and Kidney Diseases.

^i^NCHPAD: National Center on Health, Physical Activity and Disability.

^j^NYC: New York City.

### Recruitment and Pilot Implementation

Before initiating the BRIDGE trial, we pilot-tested 2 sessions with a community partner, Senior Planet, to test the acceptability and usability of program resources described above. Senior Planet is an online learning community for older adults (aged >60 years) launched via Older Adults Technology Services from the American Association of Retired Persons (AARP). The pilot sessions were advertised via Senior Planet. The participants were existing members of Senior Planet who elected to attend the virtual session based on personal interest in the subject. Older adults participated in 2 virtual pilot sessions led by a certified lifestyle coach. The 2 chosen pilot sessions were Session 7: “Energy In, Energy Out” and Session 20: “Shop and Cook to Prevent T2” after consultation with the community partner about the group’s needs. The pilot sessions were held 1-week apart.

### Survey Description

Existing implementation measures were used to develop the survey [11], and questions were adapted with input from physicians (JC and MJ) and behavior change researchers (MAS and ESR). At the close of each educational session, a link to a voluntary, self-administered, online “open survey” was provided to assess satisfaction with the material presented. Participants were also emailed the survey link along with the educational resources provided during each pilot session. The survey consisted of 28 items and was administered across 6 screens to facilitate viewing the information on smartphones. The surveys were open for 1 week after each pilot session. The surveys were designed and administered via REDCap (Research Electronic Data Capture; Vanderbilt University), a secure online survey platform. Response options included 5-point Likert scales and free text. Adaptive questioning was used by incorporating skip patterns to minimize respondent burden. Respondents were able to change answers or review them using the back button function. Usability and technical functionality were tested by having each study member fill out the survey, record how long the process took, and report any issues. Responses helped the study team refine the resource adaptation before launching the BRIDGE trial. Participants were informed that the survey would take about 5 minutes, and the purpose of the survey was explained during each session.

### Ethical Considerations

This was a quality improvement project, which the New York University Institutional Review Board classifies as “non-human subject research.” A quality assurance certification was completed by the principal investigator. There were no monetary or nonmonetary incentives for filling out the survey. No personal identifiers were collected via the survey and no unique site data was tracked via cookies, IP check, or log file analysis. Participant consent was waived because this was certified as a quality improvement initiative focused on evaluating the program rather than the participants.

## Results

### Survey Results

In total, 189 older adults attended the 2 virtual sessions. The first session had 109 participants with 38 responding to the survey (34.9% response rate). The second session had 88 participants with 30 respondents (34% response rate)—with 10 (33%) of the 30 participants having attended the first session as well. There were 95 views of the survey, but only the 68 records that have responses to the survey were included in the analysis.

The survey assessed acceptability, feasibility, and intention to change behavior. As a measure of acceptability, 68% (40/59) of respondents strongly agreed that they liked the group session (Table 2), with 62% (34/55) strongly agreeing that they would recommend the program to a family member or a friend. The virtual format was well received, with 89% (47/53) of participants strongly agreeing that attending sessions was easy. Furthermore, 53% (29/55) responded that they intend to include the information presented in their daily routine.

**Table 2:** Attendee feedback.

Statement	Strongly agree, n (%)	Somewhat agree, n (%)	Neither agree nor disagree, n (%)	Somewhat disagree, n (%)	Strongly disagree, n (%)
I liked the group session. (n=59)	40 (68)	10 (17)	4 (7)	3 (5)	2 (3)
Meeting over video was a good fit for me. (n=55)	40 (73)	8 (15)	4 (7)	2 (4)	1 (2)
The topics covered in the program addressed my needs. (n=55)	27 (49)	12 (22)	7 (13)	5 (9)	4 (7)
Attending the group session was easy for me. (n=53)	47 (89)	3 (6)	1 (2)	0 (0)	2 (4)
I was able to attend the group session without a lot of effort. (n=53)	46 (87)	4 (7)	1 (2)	0 (0)	2 (4)
I was able to understand the handouts that were provided. (n=53)	32 (60)	10 (19)	9 (17)	0 (0)	2 (4)
I was able to find the information I was looking for on the website without a lot of effort. (n=53)	24 (45)	12 (23)	13 (24)	1 (2)	3 (6)
I would recommend this program to a friend. (n=55)	34 (62)	11 (20)	4 (7)	4 (7)	2 (4)
I intend to include the information presented today in my daily routine. (n=55)	29 (53)	12 (22)	9 (16)	2 (4)	3(6)

### Resource Relevance

Participants were also asked to choose how relevant they found the resources used during the sessions: videos, group discussion, website, and handouts (Table 3). More than three-quarters of participants found the additional resources at least somewhat relevant to their lives. Participants were also asked an open-ended question about what topics they would like to see in the future. A total of 24 participants responded: 9 said they would like more focus on meal-planning, 7 said they would like to learn about how diabetes affects other organ systems, and 6 requested more advanced information on diabetes. Only 2 participants mentioned using the Healthwatch 360 (GB Healthwatch) app that was introduced in the first session.

**Table 3:** Participant perspectives of relevance of the program items.

Program resource	Very relevant, n (%)	Somewhat relevant, n (%)	Not very relevant, n (%)	Not relevant at all, n (%)	Do not recall or did not review, n (%)
Videos (n=51)	31 (61)	12 (23)	5 (10)	1 (2)	2 (4)
Group discussions (n=51)	32 (63)	16 (31)	3 (6)	0 (0)	0 (0)
Handout (n=51)	28 (55)	12 (23)	3 (6)	1 (2)	7 (14)
Website^a^ (n=24)	11 (46)	5 (21)	3 (12)	0 (0)	5 (21)

^a^The website resource was only introduced for the second pilot session.

## Discussion

### Principal Findings

This paper describes the adaptation of an evidence-based lifestyle change program aimed at preventing type 2 diabetes in high-risk individuals to better meet the needs of older adults. Participant responses were positive, indicating minimal barriers to attending sessions and a positive response to program materials. Participants also indicated that they learned things they could implement in their own lives to make healthier choices and that they would recommend the program. Participants also deemed the additional provided resources (videos, handouts, and study website) as relevant to their lives.

The DPP is a year-long program with just 67.6% of adults age ≥65 years retained after the first 18 weeks on average [12]. Measures to improve the experience of older adults and counter health care disparities [13] within the program may help improve retention and individual success at weight and hemoglobin A_1c_ reduction and prevention or delay of type 2 diabetes. Hearing and visual acuity may be lower in older adults, which can lead to isolation, reduced participation in programming, and poor health outcomes [14]. Other barriers, including social determinants of health, physiologic changes of aging, and disease related declines in health or physical functioning, may still arise and preclude older adults from participating in this or other group education programs [15]. However, the study team has made adaptations to the materials (large print and subtitles or transcripts for all videos) and to the delivery (available personal amplifiers, large print on slides, and available headphones and iPads for virtual participants) in order to reduce as many barriers as possible within the time and budgetary allotments.

In preparation for the commencement of a larger randomized controlled trial, we convened our multidisciplinary team with expertise in geriatrics (JC), behavior change (ESR and MAS), nutrition (JMB and EAJ), and weight management (MJ and MAS). The team helped to develop and refine the survey questions before pilot-testing the strategies to assess acceptability. The findings of this study were shared with the team and will be used to enhance a randomized controlled trial funded by the National Institutes of Health, comparing the effectiveness of an in-person versus virtual delivery of the adapted DPP on weight loss, hemoglobin A_1c_, and attendance [8]. Several changes were made to the curriculum based on the feedback received. For example, participants are explicitly oriented to the website and other tools within the intervention session, and the sessions are more engaging rather than didactic.

Evidence from the original DPP trial [2] showed how effective the program was in older adults. Older adults have successfully engaged in online lifestyle interventions [16], and virtual programming may help to overcome the aforementioned barriers. Regardless of delivery method, the goal of DPPs remains the same—to help participants make positive changes that encourage healthy eating, losing weight, and lowering risk of developing type 2 diabetes. The BRIDGE initiative will preserve the structural curriculum but will expand the number and quality of resources for older adults. This work will help inform the development of community and clinical programs, as currently less than 15% of rural counties and 48% of urban counties in the nation have a DPP site [17].

### Limitations

A limitation to our approach was the lack of a formal conceptual model guiding adaptations [18]. We instead relied on an iterative feedback approach, where we solicited input from study team members, potential participants, and students in shaping the adaptations. Using a more rigorous framework for adaptations would help ensure adequate incorporation of stakeholder input. We were also limited by a low survey response rate (<40%), although this is not atypical in community-based work [19]. Although some participants did struggle to mute and unmute themselves and use the Zoom chat box, participants were able to participate in telehealth-style educational sessions with minimal technological issues [20]. A scoping review found that adherence to digital technology was high if individuals could see benefits to using them and that they reduced social isolation [19,21].

### Conclusions

This work will help inform the development of community and clinical programs. The BRIDGE randomized controlled trial will inform best practices for program delivery within a large health care system to help improve the health and well-being of older adults at risk for diabetes.

## Abbreviations

AARP: American Association of Retired Persons
BRIDGE: Bringing the Diabetes Prevention Program to Geriatric Populations
CDC: Centers for Disease Control and Prevention
DPP: Diabetes Prevention Program
REDCap: Research Electronic Data Capture

## Data Availability

The data presented in this study are available on request from the corresponding author.
